# Supplementary material for: Molecular Docking and Dynamics Simulation of Protein β-Tubulin and Antifungal Cyclic Lipopeptides
Source: Molecules. 2019 Sep 18;24(18):3387. doi: 10.3390/molecules24183387 (PMC6767525; doi:10.3390/molecules24183387)
Supplement: Supplementary file 1 [file molecules-24-03387-s001.pdf]

molecules

MDPI

Supplementary Materials

Molecular docking and molecular dynamics simulation of protein β-tubulin and cyclic lipopeptides

Nubia Noemi Cob Calan[1], Luz America Chi Uluac[2], Filiberto Ortiz Chi[3], Daniel Cerqueda Garcia[4], Gabriel Navarrete-Vázquez[5], Esaú Ruiz Sánchez[1*] and Emanuel Hernández-Nuñez[4*]

1. División de Estudios de Posgrado e Investigación, Instituto Tecnológico de Conkal, C.P.97345, Conkal, Yucatán, México, nubia.cob@itconkal.edu.mx (NNCC); esau.ruiz@itconkal.edu.mx (ERS)
2. Departamento de Física Aplicada, CINVESTAV-IPN Unidad Mérida, C.P. 97310, Mérida, Yucatán, México, chiamerika@hotmail.com (LACU)
3. CONACYT-Universidad Juárez Autónoma de Tabasco, Centro de Investigación de Ciencia y Tecnología Aplicada de Tabasco, C.P.86690, Cunduacán, Tabasco, México, filiberto.ortiz@ujat.mx (FOC)
4. CONACYT-Departamento de Recursos del Mar, CINVESTAV-IPN Unidad Mérida, C.P.97310, Mérida, Yucatán, México, dacegabiol@ciencias.unam.mx (DCG); emanuel.hernandez@cinvestav.mx (EHN)
5. Facultad de Farmacia, Universidad Autónoma del Estado de Morelos, C.P.62209, Cuernavaca, Morelos, México, gabriel_navarrete@uaem.mx (GNV)

* Correspondence: *E-mail address*: esau.ruiz@itconkal.edu.mx (ERS); emanuel.hernandez@cinvestav.mx, (EHN); Tel.: +52 -999-912-4130(ext. 145) (ERS); +52-999-105-5211(EHN)

Table S1. Identification of the tubulin-B subunit in the fungal species studied.

Microorganism	Accession	Homology
Fusarium oxysporum f. sp. Lycopersisi 4287	XP_018241948.1	84%
Colletotrichum gloeosporioides Nara 5	XP_ELA34262.1	84%
Alternaria alternata	XP_018387617.1	84%
Fusarium solani f. sp. pisi	XP_003046201.1	84%

Table S2. Preserved domains of the β-tubulin in the fungal species studied.

Microorganism	Accession	Domain		E- value	
		Tubulin/FtsZ	Tubulin	Ind	Cond
Fusarium oxysporum f. sp. Lycopersisi 4287	XP_018241948.1	3-212	261-382	$5.8x10^{-68}$	$6.9x10^{-72}$
				$7.8x10^{-42}$	$9.3x10^{-46}$
Colletotrichum gloeosporioides Nara 5	XP_ELA34262.1	3-212	261-382	$9.0x10^{-68}$	$1.1x10^{-72}$
				$5.4x10^{-42}$	$6.4x10^{-46}$
Alternaria alternata	XP_018387617.1	3-212	261-382	$3.0x10^{-68}$	$3.6x10^{-72}$
				$7.2x10^{-42}$	$8.7x10^{-46}$
Fusarium solani f. sp. pisi	XP_003046201.1	3-212	261-382	$4.1x10^{-68}$	$4.9x10^{-72}$
				$3.6x10^{-42}$	$4.3x10^{-46}$

Table S3. Validation of β-tubulin (PDB=1JFF) as a receptor using Autodock-vina.

Position						The average of binding of taxol	The average of RMSD*
size X	size Y	size Z	center X	center Y	center Z		
20	20	20	0.628	-8.592	15.581	-7.0	3.163
22	22	22	0.628	-8.592	15.581	-7.7	3.168
25	25	25	0.628	-8.592	15.581	-7.5	3.396
30	30	30	0.628	-8.592	15.581	-9.4	2.359
40	40	40	0.628	-8.592	15.581	-9.6	3.494
50	50	50	0.628	-8.592	15.581	-9.5	3.149
60	60	60	0.628	-8.592	15.581	-9.5	3.535
20	20	20	-0.961	-7.767	17.322	-6.9	3.097
22	22	22	-0.961	-7.767	17.322	-6.5	3.591
25	25	25	-0.961	-7.767	17.322	-7.7	3.431
30	30	30	-0.961	-7.767	17.322	-9.4	2.386
40	40	40	-0.961	-7.767	17.322	-9.3	3.551
50	50	50	-0.961	-7.767	17.322	-9.4	3.849
60	60	60	-0.961	-7.767	17.322	-9.5	3.542
20	20	20	-2.265	-7.164	17.568	-7.1	2.890
22	22	22	-2.265	-7.164	17.568	-6.8	3.488
25	25	25	-2.265	-7.164	17.568	-7.7	3.422
30	30	30	-2.265	-7.164	17.568	-9.1	1.564
40	40	40	-2.265	-7.164	17.568	-9.5	3.388
50	50	50	-2.265	-7.164	17.568	-9.3	3.523
60	60	60	-2.265	-7.164	17.568	-9.4	3.620

* The average from ten replication for each position. RMSD is root mean square deviation

Table S4. Affinity of the molecular coupling of the best protein complexes-β-tubulin-lipopeptides.

Compound	No. of hydrogen bonds	Residues Receptor	Ligand	Bond length (Å) molecule	Docking score (Kcal/mol)
Taxol	1	Thr276(N)	O06	2.92	-9.1
Iturin A	2	Pro274(O)	(ND2)68	2.38	-7.0
		Thr276(O)	(ND2)66	2.16	-7.0
R2	1	Thr276(O)	(ND2)66	2.16	-7.0
R3	3	Thr276(O)	(OH)71	3.49	-7.0
		Thr276(OG1)	(OH)71	2.64	-7.0
		Thr276(N)	(OH)54	2.82	-7.0
Fengycin	3	Glu27(OE2)	(O)114	2.11	-7.0
		Thr276(O)	(N)115	2.43	-7.0
		Thr276(OG1)	(N)115	2.09	-7.0
R2	3	Ser236(OG)	(O)114	3.59	-7.0
		Thr276(O)	(N)115	2.72	-7.0
		Thr276(OG1)	(N)117	2.29	-7.0
R3	2	Glu27(OE2)	(O)114	1.95	-70
		Thr276(O)	(N)116	2.56	-7.0
Surfactin	1	His229(NE2)	H	2.7	-6.3
R2	1	His229(NE2)	H	2.7	-6.3
R3	1	His229(NE2)	H	2.0	-6.3

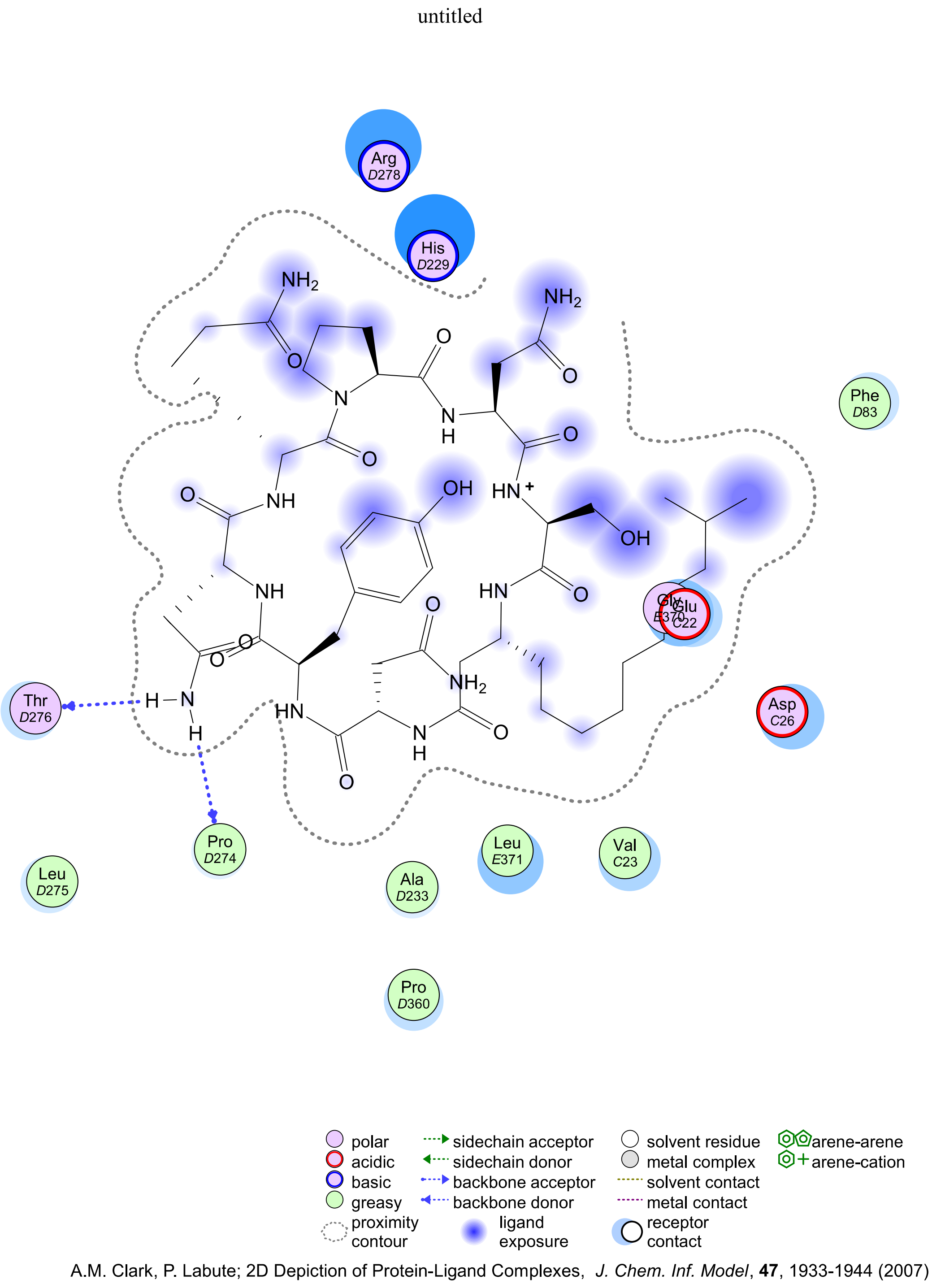

Figure S1. Interaction of docking molecular with active site β-tubulin-iturin A1.

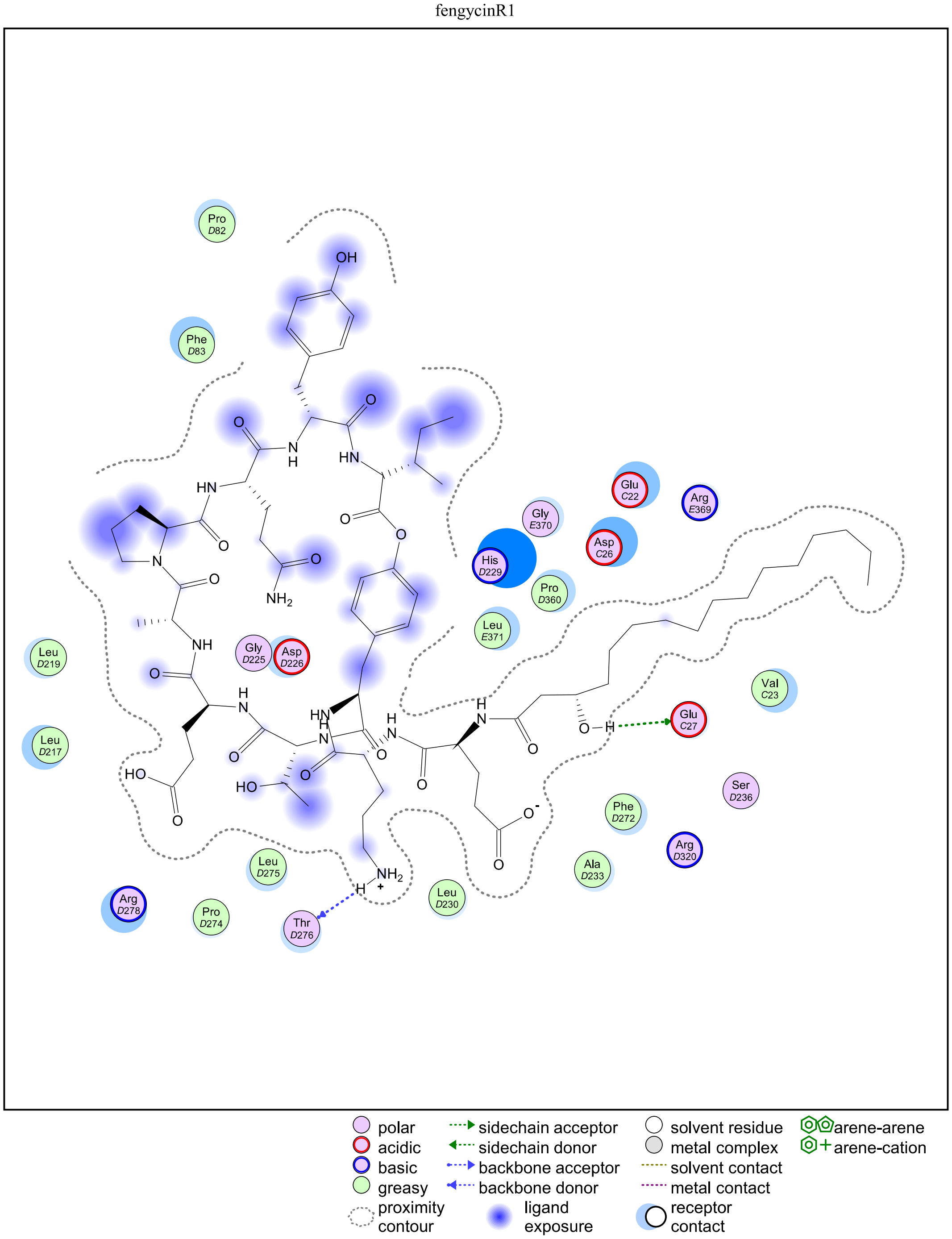

A.M. Clark, P. Labute; 2D Depiction of Protein-Ligand Complexes, *J. Chem. Inf. Model*, **47**, 1933-1944 (2007)

Figure S2. Interaction of docking molecular with active site β-tubulin-fengycin.

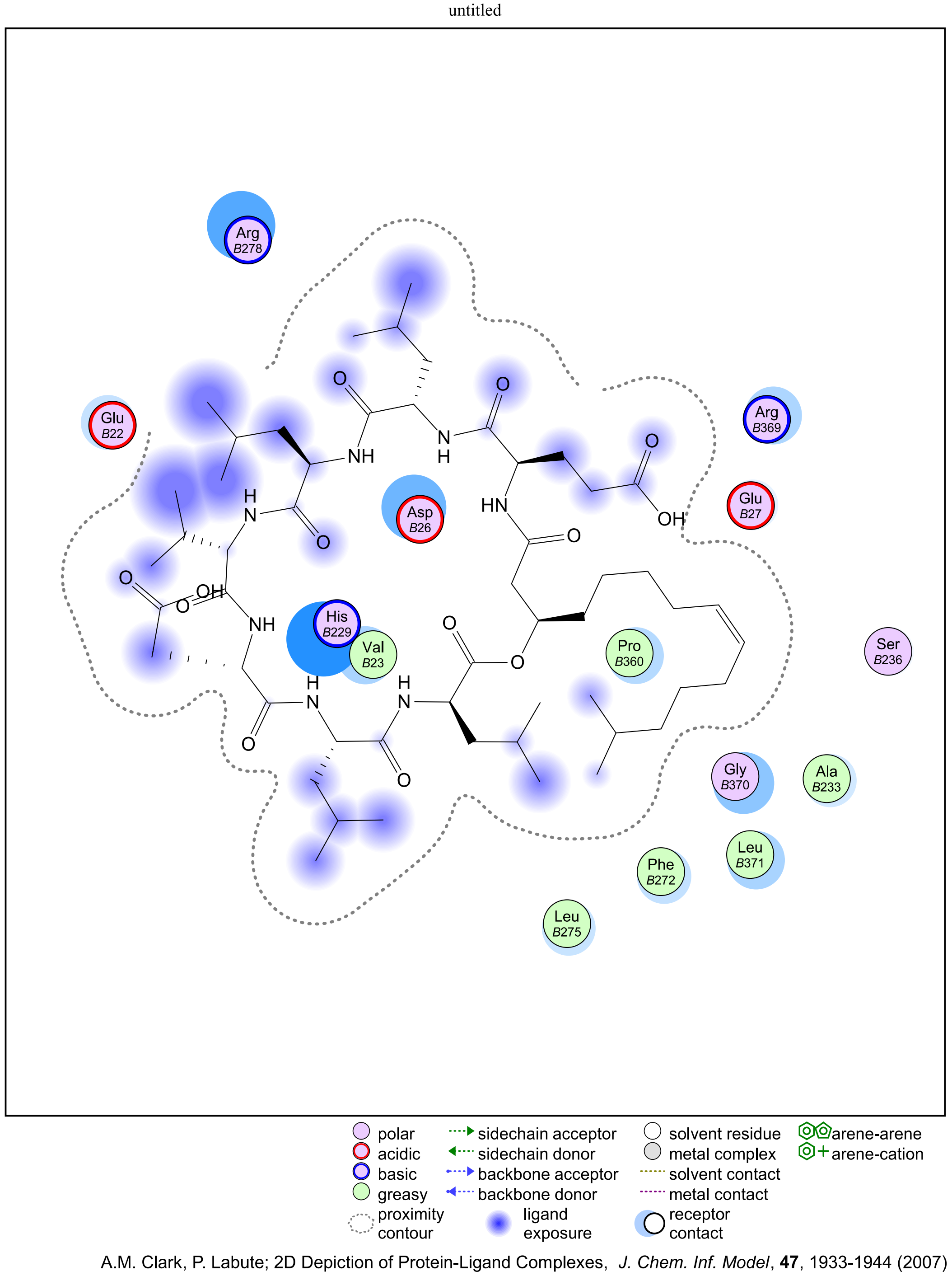

Figure S3. Interaction of docking molecular with active site β-tubulin-surfactin.
